# Association between Pulpal-Periapical Pathology and Autoimmune Diseases: A Systematic Review

**DOI:** 10.3390/jcm10214886

**Published:** 2021-10-23

**Authors:** Julia Guerrero-Gironés, Antonio Ros-Valverde, María Pilar Pecci-Lloret, Francisco Javier Rodríguez-Lozano, Miguel Ramón Pecci-Lloret

**Affiliations:** Special Care Dentistry and Gerodontology Unit, School of Medicine and Dentistry, IMIB-Arrixaca, University of Murcia, 30100 Murcia, Spain; julia.guerrero@um.es (J.G.-G.); antoniorosvalverde@gmail.com (A.R.-V.); fcojavier@um.es (F.J.R.-L.); miguelramon.pecci@um.es (M.R.P.-L.)

**Keywords:** apical periodontitis, irreversible pulpitis, autoimmune disease, type I diabetes mellitus, rheumatoid arthritis, inflammatory bowel disease

## Abstract

Several studies have linked apical periodontitis and systemic diseases. The aim of this study is to present a systematic review of the available literature investigating whether there is an association between pulpal-periapical pathology and autoimmune disease. The review was conducted following the PRISMA statement. A literature search was performed in five databases. Studies involving patients with pulpal-periapical pathology and autoimmune diseases were included in the review. Based on the PICO model, the research question aimed to assess whether there is an increased risk of developing pulpal-periapical pathology in patients with autoimmune disease. Article selection, data extraction, and quality assessment were performed using an adapted version of the STROBE guidelines. A total of seven studies were included in our review. The types of articles were five case-control and two cross-sectional studies. Periapical pathologies were associated to three autoimmune diseases (diabetes mellitus I, rheumatoid arthritis, and inflammatory bowel disease). Among the included studies, four show a low risk of bias, while three present a moderate risk. There could be an association between apical periodontitis and autoimmune diseases, although most studies report statistically non-significant associations.

## 1. Introduction

Dental caries is the most common cause of pulp and periapical pathology. When the carious lesion progresses, the pulp undergoes histological and morphological changes, causing an inflammatory response (reversible pulpitis) [1]. If the pulp is treated correctly, it reverts to its healthy state. When the pulp is unable to heal, the diagnostic term for this condition is irreversible pulpitis [2]. The acutely inflamed pulp is symptomatic, and the chronically inflamed pulp is asymptomatic [1]. As irreversible pulpitis progresses, pulp necrosis may develop. Both irreversible pulpitis and pulp necrosis are two pulp conditions that require endodontic treatment which, if not performed, can result in an extension of the lesion beyond the apex of the tooth and lead to periapical disease [1,3].

Autoimmune diseases are a series of pathologies in which the immune response to self-antigens causes damage or dysfunction in tissues by producing autoantibodies [4,5]. The increase in the incidence of these diseases in industrialized countries is indicative that environmental factors, such as lifestyle, smoking, hygiene, consumption of antibiotics, and dietary factors, can trigger autoimmunity in genetically susceptible individuals. Therefore, etiologically, there is both a genetic and environmental component [6,7].

In general, autoimmune diseases are considered relatively rare, but their effects on mortality and morbidity are high [8]. Today, it has been shown that they affect approximately 5% of the population [9] and that there are about 100 different autoimmune diseases that are difficult to classify [10]. Some of them are specific to certain organs, such as inflammatory bowel disease, type I diabetes mellitus, autoimmune hepatitis, and Sjögren′s syndrome. However, others involve numerous organs such as systemic lupus erythematosus, rheumatoid arthritis, and dermatomyositis [6,10]. Drug therapies, new evidence-based advances in molecular immunology, and gene therapy have proven themselves valuable in improving the outcome and prognosis of patients with these diseases [10,11]. Research suggests that modifiable behaviors, such as physical activity and stress, can reduce the incidence and improve outcomes of these diseases [12,13].

Currently, the scientific literature reports an association between periodontal disease and systemic diseases [14,15,16]. Although they are different entities, periodontal and periapical lesions have overlapping pathogenic mechanisms: both develop from a complex inflammatory immune response triggered by microbial elements, leading to bone destruction. The ultimate goal of treatment in both conditions is to control microbial factors, allowing the cessation of local chronic inflammatory osteolysis, which would boost the host′s response to the repair of damaged tissues [17,18].

For this reason, it can be suggested that apical periodontitis should be associated with the same systemic conditions with which periodontal disease is associated. In a similar manner, one study states that non-specific immune system integrity is a significant predictor for endodontic treatment and its subsequent outcome [19]. Other research also reported the impact of an impaired non-specific immune system on periapical tissue healing [19]. In this way, the pro-inflammatory environment and the impaired immune response associated with systemic diseases, such as autoimmune diseases, could negatively influence the reparative response of the pulp and periapical tissue [18].

Several authors support that apical periodontitis can act as a source of infection in distant sites of the body and that it can increase the symptoms caused by systemic inflammatory diseases [18,20,21], such as cardiovascular disease [22] or type 2 diabetes mellitus [23].

However, although the relationship between pulpal-periapical disease and autoimmune diseases has been studied, there is no systematic review that synthesizes the relationship between pulpal-periapical disease and all autoimmune diseases in a single article.

Accordingly, the aim of this study is to perform a qualitative synthesis of available studies that assesses the possible relationship of pulpal-periapical pathology and autoimmune diseases.

## 2. Materials and Methods

This systematic review was conducted following the PRISMA 2020 Statement (Preferred Reporting Items for Systematic Reviews and Meta-Analyzes) [24], and it has been registered in PROSPERO registry (CRD42021243260).

The research question was based on the PICO model: this review aimed to assess the possibility of a greater risk of developing pulpal-periapical pathology in patients with autoimmune disease (P: Patient with autoimmune disease; I: Presence of pulpal-periapical pathology with and without root canal treatment; C: Risk of pulpal-periapical pathology in patients with and without autoimmune disease: O: Prevalence of pulpal-periapical pathology in patients with autoimmune disease).

### 2.1. Search Strategy

The bibliographic search was carried out in February 2021 in five electronic databases (MEDLINE, SciELO, Web of Science, Cochrane Library, and Scopus). In the second phase, a manual search was performed. The structured search strategy and data extraction were conducted by an individual examiner.

The search strategy included seven uncontrolled descriptors: “autoimmune disease”, “autoimmunity”, “periapical lesion”, “apical periodontitis”, “irreversible pulpitis”, “endodontic”, and “root canal treatment”. The Boolean operators “OR” and “AND” were used to annex the search terms related to the search question (Table 1). Descriptors were searched in abstracts, titles, and abstracts of the databases. Additionally, a manual search of the references of the included studies was performed to search for potentially eligible studies.

### 2.2. Inclusion and Exclusion Criteria

Inclusion and exclusion criteria are summarized in Table 2. These were established by consensus between the authors, considering the research question and the study objectives while aiming to obtain a wide range of results from the search strategy.

### 2.3. Study Selection

The bibliographic references identified by the search terms mentioned above were exported to EndNote reference management software (Clarivate Analytics, London, UK) to check for possible duplicates. A first screening of record titles and abstracts was performed following the inclusion and exclusion criteria. Subsequently, studies meeting the criteria for the present review were assessed for eligibility via full-text screening.

### 2.4. Study Data

The following variables were recorded from each article to perform a bibliometric analysis: author and year of publication, journal, country, and institution. The following details were extracted: type of autoimmune disease, study design, study groups, diagnostic criteria for apical periodontitis, results/outcomes of interest, an association of autoimmune disease and apical periodontitis, and the analyzed variables.

### 2.5. Quality Assessment

The quality of the selected studies was performed by two examiners using an adapted version of the STROBE guidelines for rating observational studies [25]. All included studies were scored according to 11 specific criteria obtained from items 5, 6, 7, 8, 10, 12, 13, and 15 from the original checklist. Each parameter was scored as positive (√) when the requirement was met and negative (×) when not met.

From the eleven criteria, studies with 8 to 11 points were categorized as low risk of bias, 4–7 were categorized as moderate risk, and those with 3 or less were considered as high risk of bias.

Final study scores from each examiner were collected and scrutinized for discrepancies. A consensus decision resolved any disagreement between raters. Once consensus was reached for all study ratings, overall quality scores were collected by adding these criteria, with the maximum score being 11.

## 3. Results

### 3.1. Study Selection and Flow Diagram

The first search identified 163 preliminary references related to pulpal-periapical pathology with autoimmune diseases. Of these, 113 were found in MEDLINE (PubMed), 16 in Scopus, 33 in Web of Science, 1 in Cochrane, and none in the SciELO databases. Table 3 details the search strategy in the different databases used and the results found in each of them.

After excluding 10 duplicates, the remaining 153 were examined. Of these, 136 were excluded when reading the title and abstract, since they did not meet the inclusion criteria. The remaining 17 were examined by full-text screening, and five were found to be eligible for our review.

The manual identification of articles was carried out by screening the references from the previously selected articles. After the search, two additional studies were included in the systematic review.

**Figure 1:**
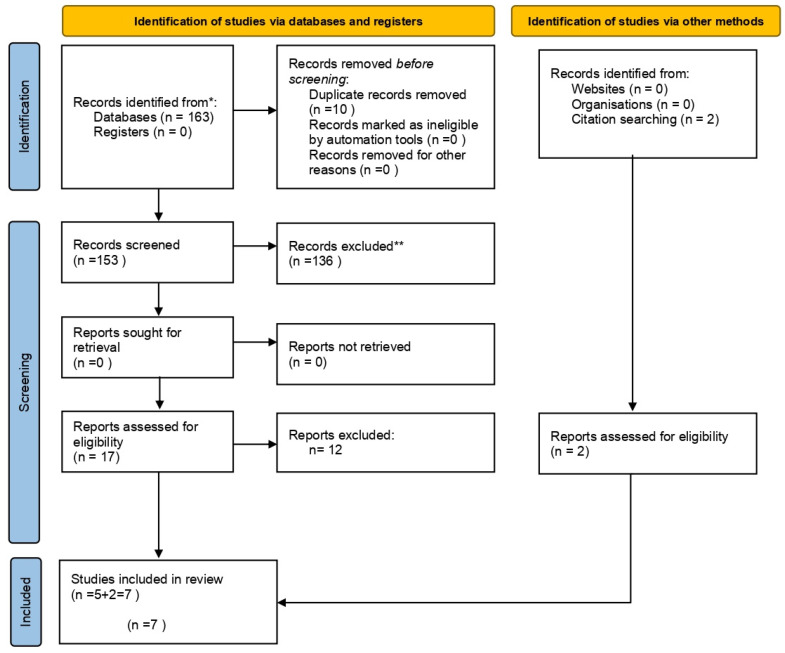
Systematic flow diagram representing the inclusion of studies according to the PRISMA 2020 Statement [24]. * Consider, if feasible to do so, reporting the number of records identified from each database or register searched (rather than the total number across all databases/registers). ** lf automation tools were used, indicate how many records were excluded by a human and how many were excluded by automation tools.

Therefore, a total of seven articles were eligible for qualitative synthesis (Figure 1).

### 3.2. Study Characteristics

#### 3.2.1. Bibliometric Analysis

**Figure 2:**
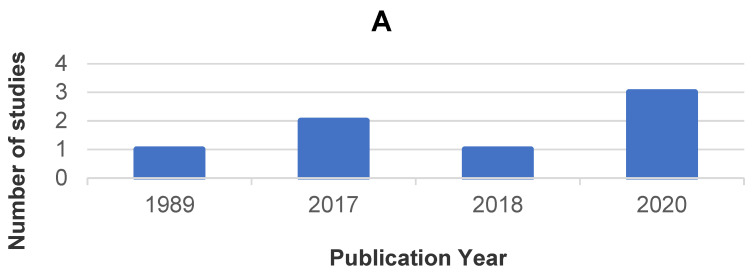

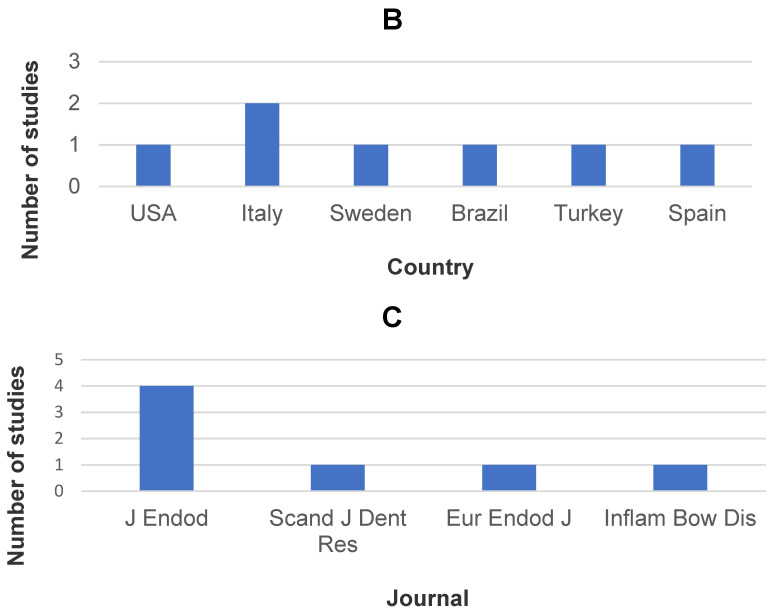
Bibliometric analysis: distribution of included studies by year of publication (**A**), country (**B**), and journal (**C**).

The distribution of the included studies by year of publication, country, and journal is presented in Figure 2.

#### 3.2.2. Type of Autoimmune Disease

This systematic review includes studies that address three types of autoimmune diseases: rheumatoid arthritis, type I diabetes mellitus, and inflammatory bowel disease. The latter can be divided into ulcerative colitis and Crohn′s disease. There are two articles (28.5% of the total articles) on rheumatoid arthritis [26,27] and type I diabetes mellitus [28,29]. Inflammatory bowel disease, on the other hand, is the most studied autoimmune disease with regard to its relationship with pulpal-periapical pathology. Three articles addressed their relationship [30,31,32], that is, 43% of the total articles (Table 4).

#### 3.2.3. Study Type

All the articles selected in the qualitative synthesis were divided into two types of studies: five were case-control studies [27,29,30,31,32], and two were cross-sectional studies [26,28]. Therefore, we can affirm that 71.5% of the studies belonged to case-control studies and 28.5% belong to cross-sectional studies (Table 4).

#### 3.2.4. Sample and Groups

From the selected studies, 100% were performed in humans, and a total of 1000 patients were analyzed. The sample size among the studies was highly heterogenous: 231 patients [29], 220 patients [32], 162 patients [27], 150 patients [28], 108 patients [30], 96 patients [26], and 33 patients [31]. Therefore, the arithmetic mean of patients in the studies is 142.85 patients. In all of the included studies, a study group and a control group were present. In four of them [26,27,30,32], half of the patients were in the study group, and the other half were in the control group. In two of the remaining studies, the proportion of patients was 2:1 in favor of the control group in one study, [28] and 2:1 in favor of the study group in the other [29]. Finally, in one study [31], 19 patients with autoimmune disease and 14 without it were assessed (Table 4).

#### 3.2.5. Diagnostic Criteria for Apical Periodontitis

All seven studies used radiographic images for the diagnosis of apical periodontitis: five used periapical radiographs [26,27,29,31,32]; four of them used orthopantomography [27,28,30,32], and one used bitewings [29]. Only three studies [26,31,32] claimed to perform a clinical examination. Finally, six of the seven studies [26,27,28,30,31,32] used the periapical index (PAI), which is the most widely used diagnostic criterion for apical periodontitis (Table 4).

#### 3.2.6. Association between Autoimmune Disease and Apical Periodontitis

Among the results from the included studies (Table 4), a statistically significant association was only found between a series of the variables in five studies. Karatas et al. [26] reported a higher prevalence of patients with rheumatoid arthritis and apical periodontitis. Cotti et al. [31] found a higher cure rate for apical periodontitis in patients with inflammatory bowel disease under treatment with biological drugs. Piras et al. [32] associated a significantly higher number of teeth with apical periodontitis in women with inflammatory bowel disease. Limeira et al. [28] described an association between diabetes mellitus and patients with apical periodontitis either before or after root canal treatment. Falk et al. [29], on the other hand, found a significant association in long-term diabetic women with root canal treatment and apical periodontitis, and the presence of two or more periapical lesions in long-term diabetic patients.

#### 3.2.7. Analyzed Variables

Each study evaluated different parameters or variables. The most relevant variables are highlighted in Table 5. The most studied variable is the gender of the patients, which was included in six studies [26,28,29,30,31,32]. In five of the studies, mean age [26,27,28,30,31] and teeth with apical periodontitis [26,27,29,30,32] were evaluated. Four studies assessed patients with apical periodontitis [26,27,28,32]. Three studies evaluated the smoking habit [26,28,30], teeth with root canal treatment [27,29,30], teeth with root canal treatment performed and apical periodontitis [27,29,30], patients with root canal treatment performed [26,27,28], and the quality of root canal treatment [27,28,31]. The variable that was least assessed was the association between patients with root canal treatment and the persistence or presence of apical periodontitis, which were assessed in only two articles [26,28].

### 3.3. Quality Assessment

All observational studies were analyzed using adapted STROBE guidelines for rating observational studies [25] (Table 6). None of the included studies was classified as presenting a high risk of bias. Four studies presented a low risk of bias, and three presented a moderate risk (57.1% of the studies presented a low risk and 42.9% presented a moderate risk). Only one of the studies [26] met all the criteria, with a score of 11. The remaining studies at low risk of bias [28,30,32] presented a score of 10, 9, and 9, respectively. On the other hand, the article with the highest risk of bias [31] obtained a score of 6 (moderate risk). The remaining studies with a moderate risk [27,29] obtained a score of 7 points (Table 7).

Regarding the criteria for the evaluation of the variables (i.e., apical periodontitis and autoimmune diseases), statistical methods, and results, all studies report or describe the methodology they used. However, one of the studies [27] did not describe the history of autoimmune disease. Interestingly, the indication of the number of participants with missing data and the explanation of how it was approached was made by only one study [26].

## 4. Discussion

Due to the possible linking factors between autoimmune diseases and apical periodontitis, i.e., they have very similar cytokine profiles (high levels of pro-inflammatory cytokines and low levels of anti-inflammatory cytokines) and the influence exerted by an altered or modulated immune system [33,34], the present study considered the possible association between the two conditions. However, there are divergent concepts regarding this association, such as those described below.

In order to combine all autoimmune diseases into a single search and perform it in a reproducible way, the search strategy was carried out using the terms “autoimmune disease” and “autoimmunity” (Table 3). There are almost 100 autoimmune diseases that are difficult to classify [10]. As a result, potentially eligible studies that could be included in the review may have gone unnoticed, as the keywords used to refer to such disease may not be present in the text of the article. This may act as a limitation of this study. In addition, with regard to the association between endodontic pathology and autoimmune diseases, only studies that assessed apical periodontitis were found. No studies on other pulp conditions (i.e., irreversible pulpitis) were found.

Regarding the prevalence of patients with apical periodontitis patients with autoimmune diseases, two studies reported that rheumatoid arthritis and type I diabetes mellitus are associated with periapical lesions: In their cross-sectional study, Limeira et al. [28] found a prevalence of patients with apical periodontitis of 58% in type I diabetics and 15% in the control group. This difference was significant (*p* = 0.011). In the study by Karatas et al. [26], 47.9% of the patients with rheumatoid arthritis presented apical periodontitis in at least one tooth, with significant differences with the control group (patients without the autoimmune disease) (*p* = 0.027). Both studies indicate that the development of apical periodontitis may be more likely in patients with rheumatoid arthritis and type I diabetes mellitus than in healthy patients. Similarly, it could be possible that an autoimmune disease could affect the success or failure of endodontic treatment. In this sense, Marending et al. claim that non-specific immune system integrity was a predictor significant for endodontic treatment and its subsequent outcome [35]. Other research also reported that a non-specific immune system impaired healing of the periapical tissue [19]. In this way, the pro-inflammatory situation and impaired immune response associated with systemic diseases, such as autoimmune diseases, could negatively influence the repair response of the pulp and the periapical tissue [18]. Jalali et al. [27], disagreeing with Karatas et al. [26], did not find significant differences between the group with rheumatoid arthritis and the control group (49.6% and 54.2%, respectively; *p* = 0.458). This disagreement may be because Jalali et al. [27] investigated the relationship of one type of apical periodontitis, rare periapical osteitis, thus ruling out the other types of apical periodontitis. Piras et al. [32] found a 64% prevalence of patients with apical periodontitis in the group with inflammatory bowel disease, while in the control group, a prevalence of 59% was found. However, this difference was not significant (*p* > 0.05).

Furthermore, Poyato-Borrego et al. [30] found a higher prevalence of teeth with apical periodontitis in patients with inflammatory bowel disease (35.2%) than in the control group (16.7%) (*p* = 0.03) [30]. In agreement with this, Karatas et al. [26] stated that that the prevalence of teeth with apical periodontitis in patients with rheumatoid arthritis is 4.3%, while in the control group, it is 2% (*p* = 0.003). However, both Jalali et al. [27] and Piras et al. [32] did not find a significantly higher prevalence of teeth with apical periodontitis in patients with this pathology (*p* = 0.0364, *p* > 0.05 respectively).

On the other hand, the prevalence of patients with at least one tooth with root canal treatment and apical periodontitis was studied by Karatas et al. [26] and Limeira et al. [28]. The first study found more subjects from the control group with this variable than in patients with rheumatoid arthritis (*p* = 0.375). However, the second did find an association in diabetic patients (*p* = 0.00). A recent systematic review [36], supporting the result of Limeira et al. [28], suggests a strong connection between the presence of periapical radiolucency in teeth with root canal treatment and diabetic patients. This fact may be related to the impaired healing ability of diabetic patients due to problems in blood circulation, response to infection, and the speed and quality of repair [37]. In addition, periapical lesions in teeth with root canal treatment may be due to persistent chronic apical periodontitis or partially healed lesions, especially when less than two years have passed after treatment [38].

Regarding gender, another recent systematic review on the prevalence of apical periodontitis in the adult population found no significant association between female and male subjects [39]. In contrast, Falk et al. [29] state that women with long-term diabetes have a higher percentage of teeth with root canal treatment and periapical lesions than short-term diabetic women or women without diabetes (*p* < 0.01) [29]. In addition, Piras et al. [32] found a higher prevalence of teeth with apical periodontitis (*p* < 0.05) and a higher prevalence of patients with apical periodontitis (*p* > 0.05) in women with inflammatory bowel disease. Both findings suggest the possibility that women with the aforementioned autoimmune diseases appear to be more likely to develop apical periodontitis. However, more evidence is needed to confirm this association. Thus, although apical periodontitis is not more prevalent in one gender than in another, in most autoimmune diseases, there are apparent differences in prevalence by gender. In fact, most often, women are more affected than men [4].

The disagreement regarding the association of apical periodontitis and different autoimmune diseases among the studies included in the present work could be attributed to the difference that exists in their methodology. An accurate diagnosis of apical periodontitis requires clinical and radiographic examinations, such as periapical or panoramic radiographs, cone-beam computed tomography (CBCT), or even a histological examination if necessary [40,41]. All the studies included in the review [26,27,28,29,30,31,32] performed radiographic studies to diagnose periapical pathology. Three studies [26,31,32] were assisted, along with other techniques, by a clinical examination to diagnose periapical disease. Without clinical confirmation, radiographic images may be insufficient for the diagnosis of apical periodontitis [42]. Furthermore, six of the seven studies [26,27,28,30,31,32] used the periapical index (PAI). Based on the proposal by Orstavik et al. [43], many other examiners have used this system for recording apical periodontitis on radiographs in different epidemiological studies [44,45,46]. This index consists of five scores ranging from 1 (healthy) to 5 (severe periodontitis with exacerbation characteristics) [43]. Only Falk et al. [29] did not use it in their study, published in 1989, but considering that PAI was proposed in 1986, it may be justified that the system had not yet been extended to researchers.

The studies included in this systematic review do not address the issue of whether the microorganisms involved in periapical lesions could somehow lead to or worsen the autoimmune disease. Nevertheless, it has been described that a high systemic inflammatory condition significantly influences the development and progression of autoimmune disease [47]. Recent studies report that although the evidence is limited, endodontic periapical infection and specific molecular markers of systemic inflammation could be closely related [21,48]. In addition, the scientific literature has examined the relationship between polymorphism and apical pathology and suggested a plausible relationship between genetic polymorphism and apical pathology [49].

Interestingly, there appears to be better and faster healing of periapical periodontitis when root canal treatment is performed in patients with inflammatory bowel disease treated with biological therapies than in healthy patients. In the study by Cotti et al. [31], the recovery rate of apical periodontitis in the study group was 100% at two years, although it began to be noticeable at 3 months, while in the control group, it was 81%, which was initially detectable from 6 months. The authors attribute this fact to a possible beneficial effect of endodontic treatment due to biological drugs. These results are in agreement with Peddis et al. [50]. Further studies are necessary, since it could be a favorable and predisposing factor for successful root canal treatment in patients with autoimmune disease taking biological drugs.

## 5. Conclusions

The analysis of the studies included in this review suggests that there could be an association between apical periodontitis and autoimmune diseases. However, it should be taken into account that the selected studies are limited, the majority report statistically non-significant associations, and some of them present a moderate risk of bias. Therefore, more prospective human studies are needed to investigate the relationship between these two medical conditions.

## Figures and Tables

**Table 1:** Search strategy.

Field 1	“autoimmune disease” OR “autoimmunity”
	AND
Field 2	“periapical lesion” OR “apical periodontitis” OR “irreversible pulpitis” OR “endodontic” OR “root canal treatment”

**Table 2:** Inclusion and exclusion criteria.

Inclusion Criteria	Exclusion Criteria
Studies including patients with pulpal-periapical pathology and autoimmune disease.	Studies that include any pathology other than pulpal-periapical in patients with autoimmune disease.
Case-control studies, cohort studies, cross-sectional descriptive studies, systematic reviews, and meta-analyses	Clinical cases
Human studies	Animal studies
Studies in English and Spanish	Studies in any language other than English or Spanish

**Table 3:** Search strategy and findings of each database.

**Search Strategy**	#1	“autoimmune disease“ OR “autoimmunity”
	#2	“periapical lesion” OR “apical periodontitis” OR “irreversible pulpitis“ OR “endodontic” OR “root canal treatment”
	#1 AND #2	(“autoimmune disease” OR “autoimmunity”) AND (“periapical lesion” OR “apical periodontitis” OR “irreversible pulpitis” OR endodontic OR “root canal treatment”)
**Database**	**Search Strategy**	**Findings**
MEDLINE	#1	494.844
	#2	47.639
	#1 AND #2	113
SciELO	#1	2.093
	#2	91
	#1 AND #2	0
Cochrane Library	#1	82
	#2	18
	#1 AND #2	1
Web of Science	#1	302.106
	#2	34.210
	#1 AND #2	33
Scopus	#1	215.584
	#2	1.185
	#1 AND #2	16

**Table 4:** Summary of main results of included studies.

Author and Year	Autoimmune Disease	Study Type	Sample and Groups	Diagnostic Criteria for AP	Results of Interest	Association AD and AP
Karatas et al., 2020 [26]	Rheumatoid Arthritis	Cross-sectional	*n* = 96 patients, 2051 teeth SG = 48 patients, 1026 teeth CG = 48 patients, 1025 teeth	Periapical XR Clinical examination Periapical Index (PAI)	≤1 teeth with AP in SG: 4.3%, in CG: 2% (OR = 2.193; *p* = 0.003)	Yes
					RCT+AP in patients in SG: 10.4%, in CG: 12.5% (OR = 0.473; *p* = 0.375)	No
					Patients with AP in SG: 47.9%, in CG: 29,7% (OR = 3,087; *p* = 0.027)	Yes
Jalali et al., 2017 [27]	Rheumatoid Arthritis	Case-control	*n* = 162 patients, 6855 teeth SG = 131 patients, 3260 teeth CG = 131 patients, 3395 teeth	Panoramic XR Periapical XR Periapical Index (PAI)	≤1 teeth with AP in SG: 3.96%, in CG: 3.53% (*p* = 0.364)	No
					≤1 teeth with RCT+AP in SG: 24.1%, in CG: 30,7% (*p* = 0.142)	No
					Patients with AP in SG: 49.6%, in CG: 54.2% (*p* = 0.458)	No
Poyato-Borrego et al., 2020 [30]	Inflammatory Bowel Disease	Case-control	*n* = 108 patients SG = 54 patients CG = 54 patients	Panoramic XR Periapical Index (PAI)	≤1 teeth with AP in SG: 35.2%, in CG: 16.7% (OR = 2.75; *p* = 0.03)	Yes
					≤1 teeth with RCT+AP in SG: 48.3%, in CG: 36.4% (OR = 1.63; *p* = 0.39)	No
Cotti et al., 2018 [31]	Inflammatory Bowel Disease	Case-control	*n* = 33 patients, *n* = 44 teeth with AP SG = 19 patients, 22 teeth with AP CG = 14 patients, 22 teeth with AP	Periapical XR Clinical examination Periapical Index (PAI)	Cure rate at 3 months in SG: 100%, in CG: 95.5% (*p* = 1.00). Cure rate at 2 years in SG: 100%, in CG: 81.8% (*p* = 0.108)	Yes, there is a higher cure rate in SG with biological therapy
Piras et al., 2017 [32]	Inflammatory Bowel Disease	Case-control	*n* = 220 patients SG = 110 patients CG = 110 patients	Panoramic XR Clinical examination: Periapical XR Periapical Index (PAI)	Patients with AP in SG: 64%, in CG: 59% (*p* > 0.05)	No
					Number of teeth with AP higher in SG than CG (*p* > 0.05)	No
					Higher risk of AP in women of SG than CG (*p* > 0.05)	No
					The number of teeth with AP was higher in SG woman than CG (*p* < 0.05)	Yes
Limeira et al., 2020 [28]	Diabetes mellitus type 1	Cross-sectional	n= 150 patients SG = 50 patients CG = 100 patients	Panoramic XR Periapical Index (PAI)	Patients with PA in SG: 58%, in CG: 15% (OR = 3.508; *p* = 0.011)	Yes
					Patients with RCT+AP in SG: 52%, in CG: 8% (OR = 7.220; *p* = 0.00)	Yes
Falk et al., 1989 [29]	Diabetes mellitus type 1	Case-control	*n* = 231 SG = 154 patients CG = 77 patients	Periapical XR Bitewing XR	Higher frequency of RCT+AP in LTD than CG	No
					Women: LTD higher % of RCT+AP than STD and non-diabetics (*p* < 0.01)	Yes, only in women
					LTD 40%, SDD 15% and non-diabetics 23% have ≥2 teeth with AP (*p* < 0.001)	Yes

AD: Autoimmune disease; SG: Study group; CG: Control group; XR: Radiograph; AP: Apical periodontitis; RCT: Root canal treatment; OR: Odds ratio; LTD: Long-term diabetic; STD: Short-term diabetic.

**Table 5:** Analyzed variables of included studies.

	Gender	Age	Smoking Habit	Teeth with AP	Teeth with RCT	Teeth with RCT+AP	Patients with AP	Patients with RCT	Patients with RCT+AP	Quality of RCT
Karatas et al. [26]	Yes	Yes	Yes	Yes	No	No	Yes	Yes	Yes	No
Jalali et al. [27]	No	Yes	No	Yes	Yes	Yes	Yes	Yes	No	Yes
Poyato-Borrego et al. [30]	Yes	Yes	Yes	Yes	Yes	Yes	No	No	No	No
Cotti et al. [31]	Yes	Yes	No	No	No	No	No	No	No	Yes
Piras et al. [32]	Yes	No	No	Yes	No	No	Yes	No	No	No
Limeira et al. [28]	Yes	Yes	Yes	No	No	No	Yes	Yes	Yes	Yes
Falk et al. [29]	Yes	No	No	Yes	Yes	Yes	No	No	No	No
Total of studies	6	5	3	5	3	3	4	3	2	3

AP: Apical periodontitis; RCT: Root canal treatment.

**Table 6:** Checklist of 11 criteria based on an adapted version of the STROBE guidelines for assessing the quality of observational studies.

**Methods**		
Setting	1	Describes the setting, participating locations, relevant dates (period of recruitment, exposure, follow-up, data collection)
Participants	2	Gives the inclusion and exclusion criteria (including paired or control groups)
	3	Describes autoimmune disease history
Variables	4	Clearly defines apical periodontitis and its diagnostic criteria
Data sources/measurement	5	Describes methods of evaluation of apical periodontitis
Study size	6	Explains how the study sample size was arrived at
Statistical methods	7	Describes statistical methods, including those used to control for confounders
	8	Describes any methods used to examine subgroups and interactions
Results		
Descriptive data	9	Provides characteristics of study participants (e.g., demographic, clinical, social) and reports on exposures and potential confounders
	10	Indicates the number of participants with missing data and explains how this was addressed
Outcome data	11	Measures and presents exposure data

**Table 7:** Results of quality assessment using an adapted version of the STROBE guidelines.

	Falk et al. [29]	Limeira et al. [28]	Poyato-Borrego et al. [30]	Cotti et al. [31]	Piras et al. [32]	Karatas et al. [26]	Jalali et al. [27]
1	✓	✕	✓	✕	✓	✓	✓
2	✕	✓	✓	✕	✓	✓	✓
3	✓	✓	✓	✓	✓	✓	✕
4	✓	✓	✓	✓	✓	✓	✓
5	✓	✓	✓	✓	✓	✓	✓
6	✕	✓	✓	✕	✕	✓	✕
7	✓	✓	✓	✓	✓	✓	✓
8	✓	✓	✓	✓	✓	✓	✓
9	✕	✓	✓	✕	✓	✓	✕
10	✕	✕	✕	✕	✕	✓	✕
11	✓	✓	✓	✓	✓	✓	✓
Total Score Risk of Bias	7 Moderate	9 Low	10 Low	6 Moderate	9 Low	11 Low	7 Moderate
